# Supplementary material for: The Systems Biology Research Tool: evolvable open-source software
Source: BMC Syst Biol. 2008 Jun 29;2:55. doi: 10.1186/1752-0509-2-55 (PMC2446383; doi:10.1186/1752-0509-2-55)
Supplement: Additional file 1 — SBRT Archive. An archive of the current version of the Systems Biology Research Tool. [file 1752-0509-2-55-S1.zip › sbrt-1.4.0/doc/developers_guide/api/sbrt/kernel/math/package-use.html]

Uses of Package sbrt.kernel.math


|  |  |  |
| --- | --- | --- |
| **Overview** \| **Package** \| Class \| **Use** \| **Tree** \| **Deprecated** \| **Index** \| **Help** | |  |
| PREV   NEXT | **FRAMES**    **NO FRAMES**     **All Classes** |  |


---


## **Uses of Package sbrt.kernel.math**

| Packages that use sbrt.kernel.math | |
| --- | --- |
| **sbrt.ext.cplex** | Provides a class that allows communication with CPLEX. |
| **sbrt.ext.glpk** | Provides a class that allows communication with GLPK. |
| **sbrt.ext.mma.algebra** | Provides classes that allow communication with Mathematica for the purposes of algebra. |
| **sbrt.kernel.fba** | Provides classes and interfaces for creating objects relevant to Flux Balance Analysis. |
| **sbrt.kernel.fba.analysis** | Provides classes and interfaces for analyzing Flux Balance Analysis data. |
| **sbrt.kernel.fba.comp** | Provides classes and interfaces for performing Flux Balance Analysis computations. |
| **sbrt.kernel.fba.proc** | Provides classes and interfaces for executing Flux Balance Analysis processes. |
| **sbrt.kernel.math** | Provides classes and interfaces for creating objects relevant to mathematics. |
| **sbrt.kernel.math.algebra** | Provides classes and interfaces for creating objects relevant to algebra. |
| **sbrt.kernel.math.algebra.proc** | Provides classes and interfaces for executing algebra-related processes. |
| **sbrt.kernel.math.geom** | Provides classes and interfaces for creating objects relevant to computational geometry. |
| **sbrt.kernel.math.optimization** | Provides classes and interfaces for creating objects relevant to solving optimization problems. |
| **sbrt.kernel.utilities.proc** | Provides classes and interfaces for performing utility processes. |
| **sbrt.shell.io.fba** | Provides classes and interfaces for reading and writing files relevant to Flux Balance Analysis. |
| **sbrt.shell.mng.algebra** | Provides classes for creating algebra process managers. |
| **sbrt.shell.mng.fba** | Provides classes and interfaces for creating Flux Balance Analysis process managers. |
| **sbrt.shell.mng.geom** | Provides classes and for creating geometry process managers. |
| **sbrt.shell.mng.utilities** | Provides classes and interfaces for creating mathematical process managers. |
| **sbrt.shell.text.fba** | Provides classes and interfaces for defining formats for objects relevant to Flux Balance Analysis. |
| **sbrt.shell.text.math** | Provides classes and interfaces for defining formats for mathematical objects. |

Classes in sbrt.kernel.math used by sbrt.ext.cplex

****Interval****
  
          This class is used to represent closed intervals
of the form [α, β], where α ≤ β.

****LinearComb****
  
          This interface is used to represent linear combinations.

****MathExpr****
  
          This interface is used to represent mathematical expressions.

****QuadraticExpr****
  
          This interface is used to represent quadratic expressions.

Classes in sbrt.kernel.math used by sbrt.ext.glpk

****Interval****
  
          This class is used to represent closed intervals
of the form [α, β], where α ≤ β.

****LinearComb****
  
          This interface is used to represent linear combinations.

****MathExpr****
  
          This interface is used to represent mathematical expressions.

Classes in sbrt.kernel.math used by sbrt.ext.mma.algebra

****LinearComb****
  
          This interface is used to represent linear combinations.

Classes in sbrt.kernel.math used by sbrt.kernel.fba

****Interval****
  
          This class is used to represent closed intervals
of the form [α, β], where α ≤ β.

****LinearComb****
  
          This interface is used to represent linear combinations.

****MathExpr****
  
          This interface is used to represent mathematical expressions.

Classes in sbrt.kernel.math used by sbrt.kernel.fba.analysis

****Interval****
  
          This class is used to represent closed intervals
of the form [α, β], where α ≤ β.

Classes in sbrt.kernel.math used by sbrt.kernel.fba.comp

****Interval****
  
          This class is used to represent closed intervals
of the form [α, β], where α ≤ β.

****LinearComb****
  
          This interface is used to represent linear combinations.

****MathExpr****
  
          This interface is used to represent mathematical expressions.

Classes in sbrt.kernel.math used by sbrt.kernel.fba.proc

****Interval****
  
          This class is used to represent closed intervals
of the form [α, β], where α ≤ β.

****LinearComb****
  
          This interface is used to represent linear combinations.

****MathExpr****
  
          This interface is used to represent mathematical expressions.

Classes in sbrt.kernel.math used by sbrt.kernel.math

****AbstractLinearComb****
  
          This abstract class is a skeleton implementation of `LinearComb`.

****AbstractLinearComb.Term****
  
          This class is a skeleton implementation of `LinearComb.Term`.

****AbstractPolynomial****
  
          This abstract class is a skeleton implementation of `Polynomial`.

****DoubleEquiv****
  
          This class is used to compare double precision numbers for equivalence.

****EquivTester****
  
          This interface is used to represent *equivalence testers*.

****HashLinearComb****
  
          This class is a concrete implemenation of `LinearComb`.

****HashLinearComb.Term****
  
          This class is a concrete implementation of `LinearComb.Term`.

****Interval****
  
          This class is used to represent closed intervals
of the form [α, β], where α ≤ β.

****IntervalComparer.Diff****
  
          Instances of this class are used to hold a variable and a pair of
corresponding intervals.

****LinearComb****
  
          This interface is used to represent linear combinations.

****LinearComb.Term****
  
          This interface is used to represent the terms of linear combinations.

****MathExpr****
  
          This interface is used to represent mathematical expressions.

****Polynomial****
  
          This interface is used to represent polynomials.

****Polynomial.Term****
  
          This interface is used to represent the terms of a
polynomial.

****VectorComparer.Diff****
  
          Instances of this class are used to hold a variable and a pair of
corresponding intervals.

Classes in sbrt.kernel.math used by sbrt.kernel.math.algebra

****Interval****
  
          This class is used to represent closed intervals
of the form [α, β], where α ≤ β.

****LinearComb****
  
          This interface is used to represent linear combinations.

Classes in sbrt.kernel.math used by sbrt.kernel.math.algebra.proc

****LinearComb****
  
          This interface is used to represent linear combinations.

Classes in sbrt.kernel.math used by sbrt.kernel.math.geom

****Interval****
  
          This class is used to represent closed intervals
of the form [α, β], where α ≤ β.

****LinearComb****
  
          This interface is used to represent linear combinations.

Classes in sbrt.kernel.math used by sbrt.kernel.math.optimization

****Interval****
  
          This class is used to represent closed intervals
of the form [α, β], where α ≤ β.

****LinearComb****
  
          This interface is used to represent linear combinations.

****MathExpr****
  
          This interface is used to represent mathematical expressions.

****QuadraticExpr****
  
          This interface is used to represent quadratic expressions.

Classes in sbrt.kernel.math used by sbrt.kernel.utilities.proc

****Interval****
  
          This class is used to represent closed intervals
of the form [α, β], where α ≤ β.

****IntervalComparer.Diff****
  
          Instances of this class are used to hold a variable and a pair of
corresponding intervals.

****MathExpr****
  
          This interface is used to represent mathematical expressions.

Classes in sbrt.kernel.math used by sbrt.shell.io.fba

****Interval****
  
          This class is used to represent closed intervals
of the form [α, β], where α ≤ β.

****LinearComb****
  
          This interface is used to represent linear combinations.

****MathExpr****
  
          This interface is used to represent mathematical expressions.

Classes in sbrt.kernel.math used by sbrt.shell.mng.algebra

****LinearComb****
  
          This interface is used to represent linear combinations.

Classes in sbrt.kernel.math used by sbrt.shell.mng.fba

****ConstraintViolationException****
  
          This class is used to indicate a constraint violation.

****ExceededLimitException****
  
          This class is used to indicate that a limit has been exceeded.

****IllegalBoundsException****
  
          This class is used to indicate the existence of illegal bounds.

****InfeasibleSolutionException****
  
          This class is used to indicate that no feasible solution exists.

****Interval****
  
          This class is used to represent closed intervals
of the form [α, β], where α ≤ β.

****LinearComb****
  
          This interface is used to represent linear combinations.

****UnboundedVariableException****
  
          This class is used to indicate that a variable is unbounded.

Classes in sbrt.kernel.math used by sbrt.shell.mng.geom

****ExceededLimitException****
  
          This class is used to indicate that a limit has been exceeded.

****UnboundedVariableException****
  
          This class is used to indicate that a variable is unbounded.

Classes in sbrt.kernel.math used by sbrt.shell.mng.utilities

****LinearComb****
  
          This interface is used to represent linear combinations.

Classes in sbrt.kernel.math used by sbrt.shell.text.fba

****Interval****
  
          This class is used to represent closed intervals
of the form [α, β], where α ≤ β.

****LinearComb****
  
          This interface is used to represent linear combinations.

****MathExpr****
  
          This interface is used to represent mathematical expressions.

Classes in sbrt.kernel.math used by sbrt.shell.text.math

****Interval****
  
          This class is used to represent closed intervals
of the form [α, β], where α ≤ β.

****LinearComb****
  
          This interface is used to represent linear combinations.

****LinearComb.Term****
  
          This interface is used to represent the terms of linear combinations.

****MathExpr****
  
          This interface is used to represent mathematical expressions.

---


|  |  |  |
| --- | --- | --- |
| **Overview** \| **Package** \| Class \| **Use** \| **Tree** \| **Deprecated** \| **Index** \| **Help** | |  |
| PREV   NEXT | **FRAMES**    **NO FRAMES**     **All Classes** |  |


---
